# Developing policy analytics for public health strategy and decisions—the Sheffield alcohol policy model framework

**DOI:** 10.1007/s10479-013-1451-z

**Published:** 2013-10-08

**Authors:** Alan Brennan, Petra Meier, Robin Purshouse, Rachid Rafia, Yang Meng, Daniel Hill-Macmanus

**Affiliations:** School of Health and Related Research (ScHARR), The University of Sheffield, Regent Court, Regent Street, Sheffield, S1 4DA UK

**Keywords:** Policy evaluation, Analytics, Modelling methodology, Public health

## Abstract

This paper sets out the development of a methodological framework for detailed evaluation of public health strategies for alcohol harm reduction to meet UK policy-makers needs. Alcohol is known to cause substantial harms, and controlling its affordability and availability are effective policy options. Analysis and synthesis of a variety of public and commercial data sources is needed to evaluate impact on consumers, health services, crime, employers and industry, so a sound evaluation of impact is important. We discuss the iterative process to engage with stakeholders, identify evidence/data and develop analytic approaches and produce a final model structure. We set out a series of steps in modelling impact including: classification and definition of population subgroups of interest, identification and definition of harms and outcomes for inclusion, classification of modifiable components of risk and their baseline values, specification of the baseline position on policy variables especially prices, estimating effects of changing policy variables on risk factors including price elasticities, quantifying risk functions relating risk factors to harms including 47 health conditions, crimes, absenteeism and unemployment, and monetary valuation. The most difficult model structuring decisions are described, as well as the final results framework used to provide decision support to national level policymakers in the UK. In the discussion we explore issues around the relationship between modelling and policy debates, valuation and scope, limitations of evidence/data, how the framework can be adapted to other countries and decisions. We reflect on the approach taken and outline ongoing plans for further development.

## Introduction

Alcohol is ‘no ordinary commodity’ and most developed countries have implemented policies to regulate its harmful effects (WHO 2011). Controlling alcohol affordability and availability are amongst the most effective policy options available to governments (Babor et al. 2010). These policies can be unpopular with consumers and industry, so it is imperative that decisions are based on sound evaluation of policy alternatives. Modelling estimates the downstream effects of policies, to show comparative effectiveness and cost-effectiveness on a range of dimensions, and enable decision makers to consider trade-offs.

The genesis of this paper was two commissioned research projects for UK policymakers. The first, for Department of Health England (Brennan et al. 2008) required systematic evidence reviews (how price/promotion links to patterns of alcohol consumption/harm, effectiveness of related policy interventions) and modelling of potential policy effects on population level health and crime outcomes (including four ‘priority subgroups’—people under 18, 18–24 year old binge drinkers, harmful drinkers who damage their physical/mental health or cause harm to others, and those on low incomes) and impacts on the alcohol industry and wider economy. The second, for the National Institute for Health and Clinical Excellence (NICE) for England and Wales (see Purshouse et al. 2009, 17), focussed on three forms of broad policy intervention:^1^ price controls including general price increases, minimum unit pricing and restricting price-based promotion (which might apply in the ‘off-trade’ i.e. supermarkets, off-licenses etc. and/or in the ‘on-trade’ i.e. pubs, bars, restaurants etc.); managing alcohol availability including regulating ‘alcohol outlet density’ and licensing hours; and advertising controls including proportions of advertising time for public health messages, eliminating under-18s television advertising, and a total advertising ban. A framework to address these questions requires up to date information on patterns of consumption and harm at national level, the ability to drill down to subgroups defined by age/sex/levels of drinking, and the functionality to project forward in time the effects of policy change on consumption and harms.

The international literature on alcohol policy modelling has a number of studies, each making several structural assumptions (Chisholm et al. 2004; Gunningschepers 1989; Hollingworth et al. 2006). Each study examines the relationships from policy change to consumption over time, and/or consumption changes to estimated harms over time. All existing modelling has been done at the population level; examining cohorts rather than individuals. One of the biggest problems is that existing modelling of the relationship between a policy and consumption has typically used a very small number of ‘drinking states’ to describe the distribution of consumption within the cohort e.g. abstention, moderate consumption, heavy consumption (e.g. Chisholm et al. 2004). This quantisation of consumption into drinking states can be problematic, particularly when using econometric analyses of the effect of price, which assume a continuous distribution of drinkers’ consumption, and existing policy models have tended to crudely apply econometric results to probabilities of transition between consumption states (Chisholm et al. 2004; Hollingworth et al. 2006). To model effects over time, models have adopted either a birth cohort (Chisholm et al. 2004; Hollingworth et al. 2006) or an age-cohort approach (Holder and Blose 1987). A birth-cohort approach considers those people born in a particular time period (e.g. 1965–1969) and models their drinking history over time. This requires longitudinal data on individuals drinking patterns which is unfortunately unavailable for the UK population. An age-cohort approach models people in a particular age band (e.g. those aged 40–44) and does not consider the issue that this age band will be made up of different constituent members at different time points in the model. The key assumption required for an age-cohort approach is that the population distribution of drinking is stable over time within an age band. This approach is more feasible than a birth-cohort approach because the cross-sectional data exists, and analysis suggests that the assumption of stability within age bands over time is largely met in the UK (Brennan et al. 2008; Kemm 2003). Existing modelling of harms has been based on literature studies reporting the mathematical relationship between levels of consumption and the risk of harm. It assumes that this relationship remains stable, so that as consumption levels change within the population one can compute the revised population level risk using the ‘potential impact factor’ (PIF), which is essentially the ratio of the weighted average revised risk and the weighted average baseline risk, as first used in the seminal ‘Prevent’ model (Gunningschepers 1989). The PIF approach can be used on risk defined in terms of disease incidence rates (Doran et al. 2008), prevalence rates (Gunningschepers 1989), and of course mortality rates. A disease incidence-based approach would require morbidity incidence data over time which is much less readily available than prevalence data. To date the PIF approach has been implemented with broadly quantised risk (i.e. consumption) states, but there are no theoretical problems in adapting it to continuous distributions of risk. Whilst these developments in the international literature have been occurring, there has been little recent modelling in the UK and no analysis to date of the alcohol policy options, such as minimum pricing, currently under consideration at national level.

This paper therefore sets out the development of a methodological framework for detailed evaluation of public health strategies for alcohol harm reduction to meet UK policy-makers needs. The methods section describes both the processes of development and the model structure. The next section covers the results framework including sensitivity analyses and validation exercises. In discussion, we consider limitations, discuss adaptability to other countries, and prioritise future developments.

## Method

### Process to engage with stakeholders, identify evidence/data and develop model structure

The central aim of our alcohol policy modelling framework is to estimate how the implementation of a policy will change, over time, outcomes of interest to policymakers and stakeholders. To do this, we need to be able to elicit relevant policies and outcomes, represent the relationship between policy (and potentially other environmental factors) and outcome, and to construct a baseline from which effects can be projected. In developing the Sheffield Alcohol Policy Model framework, a series of interlinked and iterative processes were undertaken.

**Fig. 1:**
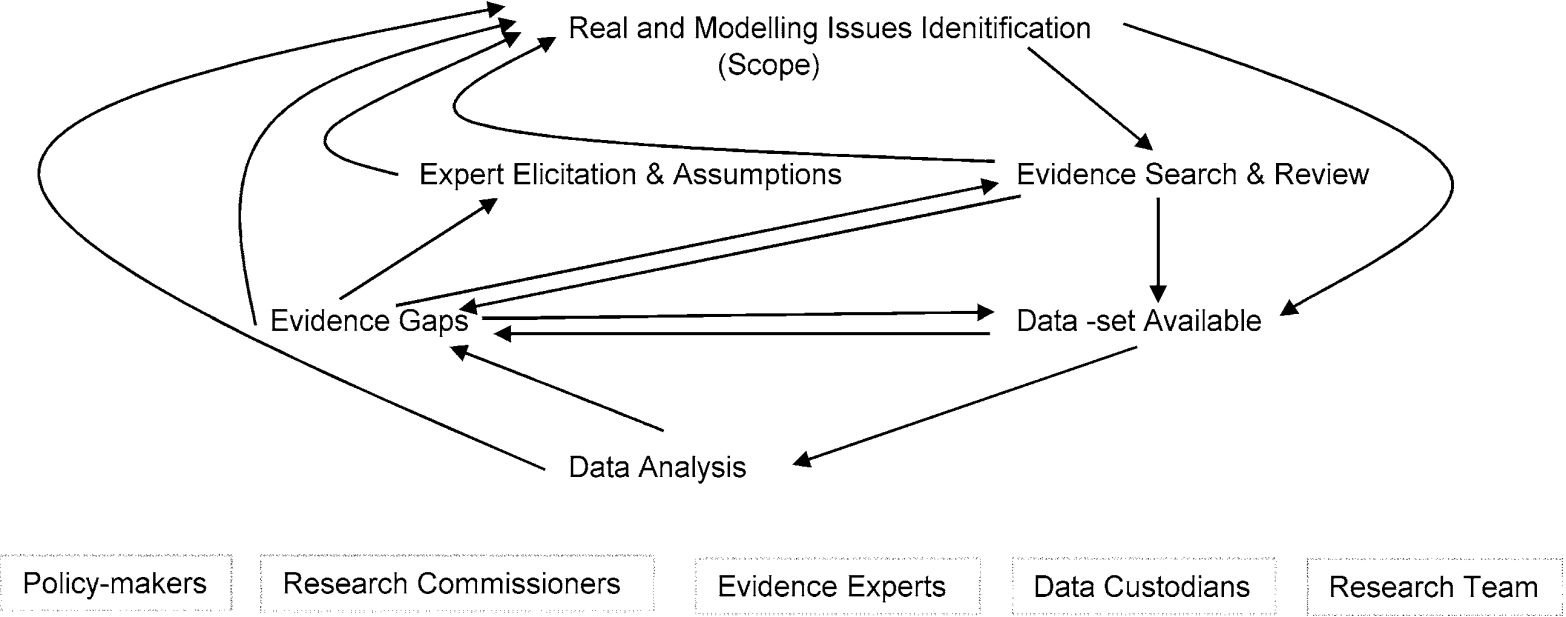
Iterative process to identify evidence, develop evaluation framework and quantified model

Throughout the process an interactive engagement continued between policy-makers, research commissioners, experts on the various domains of evidence, custodians of datasets which emerged as useful, and the research team itself (see Fig. 1). The interaction between the research team and the policy-makers/research commissioners took place with regular meetings/teleconferences in both the DH and NICE projects. These included interim written/powerpoint/verbal progress reports monthly or bi-monthly with resulting discussions on possible adjustments to scope and research plans. In some of these meetings data analyses and early model results were shared enabling participants to ask further refined research questions.

At the centre of the process was the interaction between identified issues and the systematic search for and review of published evidence. For example, the Department of Health research questions specified three systematic reviews to be undertaken (Booth et al. 2008). They covered (a) the relationship from alcohol price to consumption or directly to alcohol-attributable harm, (b) the relationship between advertising/promotion to alcohol consumption or harm, and (c) a review of reviews on the relationship between alcohol consumption and alcohol-attributable harms (appropriate because there already existed considerable research and a number of recent reviews in the area). The resulting 243 page report was a key resource in developing the modelling framework e.g. identifying two recent meta-analyses of international price elasticities by Gallet (2007) and by Wagenaar et al. (2009), which provided information on both possible methods and potential model parameter estimates. A similar approach of targeted systematic reviews agreed with policymakers/research commissioners was undertaken for the NICE project (Jackson et al. 2009, 2009b).

The comparison of issues for analysis with evidence available led to the identification of evidence gaps which in turn were pursued by searching for datasets which the team could analyse to develop evidence, discussion with experts and further literature searches. The iterative search for data sets available on the key components of the system, and early analyses of these data sets enabled the modelling team to evaluate what would be possible in terms of integrating the published evidence and the UK available data. Policy-makers and research commissioners were also involved in this process, particularly regarding where special data analysis exercises by government departments or the potential purchase of access to commercial datasets. As thinking developed, the analysis of evidence and data-sets fed back into identifying more detailed issues to address e.g. exact definitions of the different metrics for outcomes (e.g. defined sets of crimes), with this in turn followed by a further cycle of evidence and data review. In some cases evidence gaps remained after iterative searching. These factors cannot be ignored just because the evidence base is limited, and a process of considering assumptions by the research analyst or by eliciting information from experts was undertaken.

Decisions concerning whether to go into further detail in the modelling, when to elicit further expert opinion, or undertake specific sensitivity analyses were made by balancing principled and pragmatic considerations: on the one hand ‘should particular issues or details be included in principle?’, and on the other, ‘what is the likelihood of additional work affecting model results substantively?’. Here, it was fundamentally important to have clear research questions for the model to answer—one cannot prioritise further modelling effort without having at least an implicit metric for assessing the difference further detailed modelling would make to decisions.

Policy-makers and research commissioners were particularly important in iterative discussions on three aspects of the research scope. The first concerned the definitions of population subgroups for which separate results would be necessary. More details are given later, but one example was the decision, some way into the Department of Health project, to incorporate ‘moderate drinkers’ within the model, thus enabling a whole population analysis not just a focus on the four original subgroups, and the related decision to drop a separate focus on low income groups as it became clear that differential evidence for this subgroup was limited, and that the resources required to model both low income and moderate drinkers separately were beyond the project budget. The second aspect of scope concerned refined definitions of the exact policies to be tested, as both early results and understanding of the potential for implementation developed. The third concerned which dimensions of effect were most important to quantify and how each should be valued. A partial cost-benefit analysis approach has been taken, with monetary valuation of population health effects (QALYs), NHS direct costs, monetary valuation of crime victim QALY effects, criminal justice system costs, work absence and unemployment effects valued at average salary rates. Again, more detail is given later, but key decisions limiting scope of effects included: analysing alcohol industry changes only in terms of retailer income and not subsequent knock-on effects through the supply chain, analysing wider economy effects only in terms of work absence and unemployment, and excluding analysis of drinker benefits/utility/consumer surplus.

A set of what we now consider generic components for the evaluation of public health strategies emerged from these processes. In the next sections we go through each component in turn describing how it has been addressed in Sheffield Alcohol Policy Model framework.

### Classification and definition of population subgroups of interest

Three influences led to our final subgroup definitions. The first was the research brief, which implied the need to define age groups, ‘binging’ and ‘harmful’ drinking because of a request to focus on underage drinkers, 18–24 year old binge drinkers and harmful drinkers. The second was the availability of data on levels of consumption and purchasing of alcohol. Here, two key sources of data emerged from our searches. The General Household Survey (GHS) collects cross-sectional representative sample data annually for around 7,000 individuals on average weekly alcohol volume consumed over the previous year (mean weekly consumption) and maximum volume consumed during any one day during the past week (peak day consumption) (Office for National Statistics: Social and Vital Statistics Division 2008). Consumption estimates are split by type of alcohol. The GHS contains no information on prices paid or purchasing patterns. The Expenditure and Food Survey (EFS) is another annual cross-sectional household survey (different individuals from those in GHS) using a 14 day diary to record household purchases including all alcohol items purchased, the type of alcohol, the place of purchase (on-licensed vs off-licensed sector) and crucially the price paid per volume of product (Office for National Statistics and Department for Environment, Food and Rural Affairs 2008). The third influence was indirectly due the types of harms to be examined and the metrics for their valuation. The UK government had previously examined harms attributable to alcohol in terms of health e.g. lives lost, as well as crimes committed and costs to employers (Health Improvement Analytical Team 2008). Since the years of life lost if someone dies as a consequence of their alcohol consumption are related to their age, and since employment costs are related to workforce participation, we felt it necessary to extend the age-sex groupings to a broader set than those implied by the original research funder questions.

Eighteen age-sex subgroups were defined as males/females with age bands 11–15, 16–17, 18–24, 25–34, 35–44, 45–54, 55–64, 65–74, and 75+. Each age-sex group is further split into 3 drinking level groups defined as: moderate drinkers with an alcohol intake within the UK government’s recommended limits, defined as 168 g/week or less for men and 112 g/week or less for women; hazardous drinkers, defined as exceeding those limits but drinking less than 400 g/week for men and 280 g/week for women; and harmful drinkers with a weekly intake of more than 400 g/week or 280 g/week for men and women, respectively. (The UK defines a ‘unit’ of alcohol as 10 millilitres, approximately 8 g, of ethanol). This resulted in 54 defined subgroups to be modelled. Individuals in the GHS sample were also classified as a ‘binge drinker’ or otherwise based on the maximum intake of alcohol during one day of more than twice the recommended daily limit i.e. more than 64 g/day for men and more than 48 g/day for women. Note that, although the drinker subgroups are defined in terms of level of drinking i.e. moderate, hazardous and harmful, several components of the model work on an individual level basis and we actually have a continuum of mean weekly consumption specified by the individuals within the GHS.

### Identification and definition of harms and outcomes for inclusion

For health harms, our literature review quickly identified recent UK work on the health harms attributable to alcohol (Jones et al. 2008). This used routine data sources to estimate, for each person in the population according to their age group and gender, the risk of mortality and hospitalisation related to 47 different alcohol-attributable health conditions. Conditions were classified as wholly attributable to alcohol (no cases would exist in the absence of alcohol consumption, for example alcoholic liver disease) or partially attributable to alcohol (a proportion of cases would be avoided in the absence of alcohol, e.g. throat cancer). Conditions were also classified as chronic or acute, depending on whether the condition typically arises through long-term overconsumption (e.g. liver cirrhosis) or can arise through overconsumption on a single occasion (e.g. falls or road traffic accidents). Modelling of both mortality and hospitalisation rates later enabled assessment of Quality Adjusted Life Year (QALY) effects and hospital costs. Further systematic review on health harms found few conditions to add, and we decided to build the modelling of health harms to the individual on this detailed foundation. Wider harms were discussed in the literature, but were less well evidenced with little quantitative data (e.g. ‘passive’ heavy drinking effects on partners and children) and were excluded due to limited resources for primary research within our project.

Our systematic reviews further identified recent government work on crime, work absence and unemployment harms related to alcohol (Cabinet Cabinet Office/Strategy Unit 2003; Health Improvement Analytical Team 2008; Home Office Research 2011). For crime harms, analyses by the UK Home Office examined 20 classifications of crimes which can be grouped broadly into violent disorder, wounding, assault without injury, vehicle related thefts, burglary/robbery/other theft and criminal damage. Numerical estimates of total crimes reported were supplemented with suggested multipliers to account for under-reporting (the ‘multiplier’ for ‘less serious wounding’ was important in sensitivity analyses). To apportion crimes into our population subgroups, we used separate routine data on the distribution of offenders found guilty or cautioned in 2003 (Home Office 2005) for the age groups 10–15, 16–24, 25–34, 35+ for 7 offence categories, making assumptions about the mapping between these 7 offence categories and our 20 crime classifications, splitting the 16–17 from the 18–24 year olds (assuming equal probabilities) and disentangling the 10 year bands for those 35+ (assuming linearly decreasing crime rates with age).

For workplace harms, the government had examined work absence, unemployment and lost outputs due to early death (Health Improvement Analytical Team 2008). We excluded the latter to avoid double counting the social value of life years lost which we already modelled in health and crime harms. For absence, we initially planned to use the ‘Whitehall 2’ civil servants study (Cabinet Cabinet Office/Strategy Unit 2003) containing data on alcohol consumption also absence from work ‘due to injury’ or ‘for all reasons’. However this had an endogeneity problem. On the one hand, people who drink heavily might be more absent from work (causal), but on the other hand, those absent with significant illness may be less likely to drink alcohol (unrelated)—and this latter appears to be the dominant factor in that dataset. Instead we used an Australian study (Roche et al. 2008) which explicitly asked respondents whether their absence was caused by alcohol, to quantify a relationship between reported absence days caused by alcohol and level of alcohol consumption itself. For unemployment we used the same evidence as the previous government reports (Cabinet Cabinet Office/Strategy Unit 2003), i.e. a study (MacDonald and Shields 2004) which examined Health Survey for England data for males aged 22 to 64 and found that being a ‘problem drinker’ (defined using psychological/physical symptoms or quantity/frequency of consumption), reduced the probability of being in work by 6.9 %. We assumed the same figure for females but adjusted taking account of differential work participation rates by age-sex group.

### Classification of modifiable components of risk and their baseline values

The key modifiable risk factor is level of alcohol consumption for individuals and, following a review of available datasets measuring consumption, we utilised the General Household Survey (GHS, Office for National Statistics: Social and Vital Statistics Division 2006) to provide the baseline. For each sample individual, weighted at the household level to be representative of a proportion of the population, we accessed details on mean weekly consumption of standard alcohol units (enabling grouping into moderate, hazardous and harmful), and maximum per day intake in the survey week enabling a proxy analysis of binge behaviours (14,289 individuals excluding outliers). We split consumption into 4 beverage categories because prices might change differentially under different policies: beers, wines, spirits and ‘Ready-To Drinks’ (RTDs or ‘alcopops’). Data for those aged 11–15 came from the school-based Smoking Drinking and Drug Use (SDD) Survey (REF) which used the same consumption definitions as GHS and assumed individuals had equal sample weight.

### Specification of baseline position on policy variables (prices, availability, advertising)

The main focus of our policy analyses using the framework to date has been pricing. The key data-set on prices paid by subgroups is the Expenditure and Food Survey. The annual EFS (Office for National Statistics and Department for Environment, Food and Rural Affairs 2007) records a purchasing diary over a 2 week period for around 7,000 individuals in UK households. For non-durable goods, including various categories of alcoholic beverage, it records the amount of money spent (in pence), quantity purchased (e.g. in litres) and type of outlet where purchased (e.g. supermarket). We are therefore able to classify purchasing into the 4 categories of beers, wines, spirits and alcopops. Because evidence from literature suggested that price elasticities might vary by place of purchase and quality of product, we further split the beverage types into on-trade and off-trade, and into a lower-priced and higher-priced, making a total of 16 defined beverage types. We converted purchased volumes of beverage into alcohol units, using percentage alcohol by volume (%ABV) assumptions derived from sources including the ONS (Goddard 2007). We analysed anonymised individual EFS diary data, at purchased item ‘transaction’ level, (obtained via request to the government Department for Environment, Food and Rural Affairs), for 69,618 individuals, of whom 44,150 purchased alcohol over 5 years 2001/2 to 2005/6, accounting for inflation using RPI inflators for alcoholic beverages. We found discrepancies between the distribution of purchase prices from the EFS and higher level data, which we obtained from market research companies on alcohol sales of beers, wines, spirits and alcopops in the off-trade (AC Nielsen) and on-trade (CGA Strategy). EFS reported data had marginally lower mean prices (i.e. a higher proportion of cheaper alcohol) than the actual sales data. We therefore used linear interpolation to adjust the individual level EFS data so that the adjusted cumulative price distribution matched the actual sales data price distribution at 10 specified price points. With this data, we were therefore able to examine in detail the types and prices of beverages purchased by each of the 54 different population subgroups (for detailed examples see Table 1 in Purshouse et al. 2010b).

To analyse the existing extent of price promotion discounting, we obtained further analyses of market research data for both the off-trade and on-trade via procurement from AC Nielsen and CGA Strategy respectively. The underpinning data was at stock-keeping unit (SKU) level e.g. a specified branded pack of 4×300 ml bottled beer. Price information is held weekly and, using a conventional definition of discounting as a less than four week price reduction for a SKU, our suppliers were able to analyse the ‘usual price’ and the ‘sold price’ and construct aggregated summaries of discounting. This was done in 10 defined price per unit of alcohol bands enabling estimates, for example, of the volume of off-trade beer with a usual price of 35–40 p per unit which is actually sold at 30–35 p, 25–30 p etc. We were then able to use these patterns of discounting to estimate the effect of policies that restricted discounting to a certain level (e.g. a total ban on off-trade discounting, or banning buy-one-get-one free but allowing five-for-the-price-of-four, etc.).

For availability and advertising analyses, we found an evidence/data gap regarding publicly available national level data on outlet densities, opening hours and volume of advertising/marketing effort. It was not possible to establish a robust baseline for England for any of these factors, or their relationship to current patterns of consumption in England. In the next section we discuss how a high level relative change approach has been used to give policy-makers some guidance on the potential scale of effects.

### Estimating effects of changing policy variables on risk factors

In order to estimate the effect of a pricing policy on an individual’s alcohol consumption the model used an econometrics model which was derived using the adjusted EFS dataset. The econometrics model is a system of simultaneous equations relating consumption to price for the 16 modelled beverage types and also the consumption of other non-durable goods. Covariates were included for gender, age group, ethnicity, education, geographical region, household composition, household size, income and employment status. The coefficients for this system of simultaneous equations were estimated using an iterative three-stage least squares regression. Due to the volume of data available, it was found that the estimates would only converge satisfactorily for two major subgroups; (i) moderate drinkers aged 16 and over, and (ii) hazardous and harmful drinkers aged 16 and over. Finally, in order to construct the 16×16 elasticity matrices required by the Sheffield Alcohol Policy Model, the coefficients of price in the system of equations were combined to give both own price and cross-price elasticity estimates. Each elasticity estimate is the expected percentage change in consumption given a 1 % change in price. The shift in the price at which the alcohol is available paid is assumed to be defined by the policy, for example, a minimum price of 50 p per unit policy would imply all prices currently below 50 p per unit rising to exactly 50 p per unit. The econometrics model is described in further detail in a Lancet online appendix (Purshouse et al. 2010a).

It was not possible to apply this approach in order to estimate peak consumption price elasticity due to the absence of adequate data relating peak consumption to purchasing. The alternative approach that we adopted is based on the observation that in the GHS the probability and scale of peak/binge drinking is related to the mean weekly consumption. Separate linear models were estimated for each drinker type (moderate, hazardous and harmful) while the coefficient of mean consumption was allowed to vary for each gender and age group.

For advertising policies, including the use of positive public health messages, eliminating exposure of under 18’s to advertising and a total ban, only a small number of studies provide elasticity estimates relating advertising expenditure to changes in alcohol consumption. Elasticities were selected from the literature (see detail in Purshouse et al. 2009 report to NICE) and the resulting relative change in consumption is applied to all simulated individuals affected. For availability policies, including changes to the density of outlets and restrictions in hours of sale, again, elasticities were selected from the limited literature (see detail in Purshouse et al. 2009 report to NICE) and the resulting relative change in consumption applied to all simulated individuals affected. The availability analyses were of a high level ‘what-if’ kind (e.g. what if outlet density were reduced by 10 %) because detailed policies relating to outlet licensing are implemented at a local level often in tandem with other interventions such as server training, whilst national level policies are usually legislation based and act as an enabler for local action.

### Risk functions relating risk factors to harm

An epidemiological approach was used to model the relationship between consumption and harm, relating changes in the prevalence of alcohol consumption to changes in prevalence of harmful outcomes. Five inter-related concepts are involved: the total absolute level of harm occurring in the population, the alcohol attributable fraction, absolute risk functions relating the risk of harm to the level of alcohol consumption, relative risk functions relating the relative risk (RR) of harm to the level of alcohol consumption, and the potential impact fraction which calculates the change in harms following a change in consumption levels.

We categorised health harms for 47 ICD diagnosis defined conditions into 4 types: chronic harms related to mean alcohol consumption and acute harms related to peak day consumption, with a further split into conditions wholly or partially attributable to alcohol.

For chronic illnesses that are partially attributable to alcohol (e.g. oesophageal cancer), we were able to take evidence directly from published literature which provided continuous risk function curves relating mean weekly alcohol consumption in units to an individual’s relative risk (RR) of mortality or disease prevalence differentiating by sex when available (Corrao et al. 2004; Gutjahr et al. 2001; Hamajima et al. 2002; Rehm et al. 2004). We assumed that the relative risk curves are the same for each age group but that absolute risk levels differ for each age/sex group. We have direct published data on these mean absolute risks for each age sex group at baseline (e.g. annual mortality rate for oesophageal cancer for males aged 45–54) and therefore we have enough information to compute the change in absolute risk for the subgroup when the distribution of consumption changes following a policy. This change in absolute risk is calculated by multiplying the baseline absolute risk by the potential impact fraction (PIF) (Gunningschepers 1989).

1 $$ \mathit{PIF}=1-\frac{\sum_{i=0}^N w_i \bar{\mathit{RR}_i}}{\sum_{i=0}^N w_i \mathit{RR}_i} $$

where *w*_*i*_ is the sample weight for observation *i* (e.g. an individual sample from the GHS) and *N* is the number of samples in the age/sex subgroup, *RR*_*i*_ is the relative risk of harm for observation *i* given that individual’s baseline consumption level, and is the modified risk for individual *i* given their new consumption level following the policy change.

For the other three types of harm, published continuous risk function curves were not available and we developed a method to derive our own continuous risk function curves from the broader data that were available. Under this method we developed two-part linear risk functions whereby the risk is flat from zero consumption up to a particular threshold and then rises linearly as consumption increases. For each harm and age/sex subgroup it was therefore necessary to decide on an appropriate threshold after which the risk function begins to rise, and then to estimate the slope of the rising straight line risk function beyond that threshold to fit the available observed data. The central idea is that we know the distribution of alcohol consumption in England (from the GHS), we have or can derive estimates of alcohol attributable fraction (AAF), and therefore it is possible to fit a linear risk function that implies the same AAF as that observed. For acute health harms that are partially attributable to alcohol (e.g. fatal road traffic accidents), published evidence did exist on the alcohol attributable fraction (AAF) (e.g. 37 % of fatal road traffic accidents for men aged 25–34 are attributable to alcohol) (Jones et al. 2008). We assumed RR is a function of peak daily consumption, with **RR**=1 below a threshold of 3 units for women and 4 for men, and then estimated the slope of the risk function beyond the threshold by fitting the slope using ordinary least-squares regression to minimise the difference between the implied predicted AAF (the implied risk for those GHS samples with consumption above zero divided by the implied risk for the whole subgroup) and actual observed AAF for the age/sex subgroup.

For health harms wholly attributable to alcohol, a very similar approach was taken. For acute health harms which are wholly attributable to alcohol the AAF=1 by definition, and we used a similar two part linear risk function approach instead using the total observed volume of incidents (e.g. annual mortality rate for accidental poisoning by exposure to alcohol) as the metric to fit the slope of absolute risk functions in each age/sex subgroup. For chronic diseases wholly attributable to alcohol, we used the same two-part linear approach except that the risk function is related to mean weekly consumption with an assumed threshold for the start of rising risk of 2 units per day for women and 3 for men. Having estimated our own continuous risk functions for each of these types of harm, the change in risk implied by a consumption change following a policy can be calculated using the PIF.

Finally, for the chronic illnesses, debate exists about the time lag between change in exposure and change in risk (Norstrom and Skog 2001). We chose a linear lag function of 10 years to realisation of full effect i.e. the full estimated reduction in risk only occurs after 10 years and in the years between the risk reduction is linear so that at year 1 it is 1/10th of full effect, at year 2 it is 2/10ths etc. The 10 year assumption is consistent with average estimates from the literature (Norstrom and Skog 2001) and was varied in sensitivity analyses.

Crime was assumed to be partially attributable to peak alcohol consumption and we used the same approach to fit two-part linear RR functions as for partially attributable acute health harms. The AAF for every category of crime was derived from the Offending Crime and Justice Survey (OCJS) for England and Wales in 2005, using the questions which ask convicted offenders whether they had undertaken the offence because they were drunk (Home Office Research 2011). RR functions were estimated for males and females and for two age groups, under 16 and between 16 and 25, separately from the OCJS. The same RR functions were used for over 25’s based on those for 16–25, which is a limitation, but may not greatly impact on the modelling results as over 25 years old contribute to less than 30 % of all alcohol attributable crimes. Again, the change in absolute risk following change in consumption is calculated using the PIF.

Unemployment was assumed to be partially attributable to alcohol and only apply to people who drink to a harmful level based on mean consumption. It was further assumed that there is no time delay between changes in prevalence of consumption and the changes in the risk of not working. We used the same two-part linear approach to fit unemployment RR functions, using a mean consumption threshold of 5 units per day for women and 7.1 for men (i.e., thresholds defining harmful drinkers). The AAF for unemployment was estimated based on the assumption of reduced probability of working for “problem drinkers” of 6.9 % (MacDonald and Shields 2004) which was also used by the Cabinet Office in the UK to estimate the impact of alcohol misuse on unemployment (Cabinet Cabinet Office/Strategy Unit 2003). We then used the change in mean consumption to adjust observed absolute unemployment figures (from the Labour Force Survey 2006) using the PIF.

Absenteeism was assumed to be partially attributable to alcohol and relate to peak alcohol consumption, again using the two-part linear approach to fit RR functions for each age/sex group. The AAFs for absenteeism were derived from the Australian study (Roche et al. 2008); the only one identified which examined the causal relationship between alcohol and absence from work. The change in peak consumption then adjusted the baseline number of absent days for each age/sex subgroup (from the Labour Force Survey 2006) using the PIF.

### Monetary valuation

The first dimension of monetary valuation of the effects of policies is the direct financial effects of policies on consumers’ spending, retailer and government revenues when alcohol purchasing patterns change. Using the EFS purchasing data the model is able to estimate an overall change in the volume of sales (in units of alcohol) for beers/wines/spirits/RTDs to each age/sex/drinking level subgroup and the associated value of sales (in £s). When modelling price rises the level of consumer spending typically increases, and we model the overall change in retailer income separately for on-trade and off-trade but not by different particular named or types of retailer. To assess changes to government revenues, the sales value for each beverage type is apportioned into money to the retailer, alcohol duty to government and value-added tax (VAT). The average rate of duty per unit of alcohol for each beverage type was derived from work conducted by the Department of Health (Babor et al. 2010), VAT was assumed to be 17.5 %. The knock-on effects within the alcohol supply chain to manufacturers, transport companies, growers etc. is not modelled.

The second dimension of monetary valuation concerns the health, crime and workplace harms. The annual cost health harms includes the direct cost incurred by the NHS, through providing treatment or services, and also the monetary valuation of the change in population quality of life using a value for a quality adjusted life year (QALY). A Department of Health report (Health Improvement Analytical Team 2008) provided the annual NHS cost of treating most diseases attributable to alcohol, with most conditions broken down by type of consultation/service though we had to apportion some costs using the expert opinion of clinical colleagues. For diseases not covered by this report, we derived cost estimates using the average tariff from the NHS reference costs and the number of hospital admissions from Hospital Episode Statistics using the NWPHO report (Jones et al. 2008). Health related quality of life measures for each condition were extracted from the Health Outcomes Repository database (Health Outcomes Data Repository 2011) which measures QALYs using the EQ-5D around 6 weeks after hospital discharge. Following direction from the Department of Health, a quality adjusted life year was valued at £50,000 and discounted at the standard rate of 3.5 %, while health care costs were discounted at 1.5 %.

The unit cost of a crime, which includes anticipation of crime and the cost to the justice system, was taken from Brand and Rice (2000) and Dubourg et al. (2005). The harm to the victim of a crime was also taken into account through the impact on quality of life (Dolan et al. 2005), assuming the financial value of a crime victim QALY in this case to be £81,000 (Carthy et al. 1998). Additional costs include lost economic output of the victims, through not attending work and costs to the health service.

The valuation of workplace harms, which includes absence due to sickness and unemployment, were quantified based on average earnings in each age/sex group. Although costs to the public sector could also include unemployment benefit payments, these were not included due to debate as to whether these resources are lost or are in fact redistributed (Office for National Statistics 2011).

### Model structure to integrate harms analysis (modelling event histories)

The model is a hybrid model mostly operating at the resolution of population sub-group level with 54 sub-groups defined by sex, age and baseline alcohol consumption level. The vast majority of the model parameters are defined at this level. However, parameters relating to the alcohol consumption distributions (both mean daily and maximum daily) and alcohol purchasing distributions (across 16 categories of beverage) have a much more detailed level of resolution, being defined in terms of the sample individuals from the GHS or sample transactions from the EFS respectively. These samples essentially describe empirical non-parametric distributions of consumption and purchasing. Adjustments to both prices and consumption are made at the individual sample level, whilst effect sizes are calculated at an aggregate i.e. subgroup level.

**Fig. 2:**
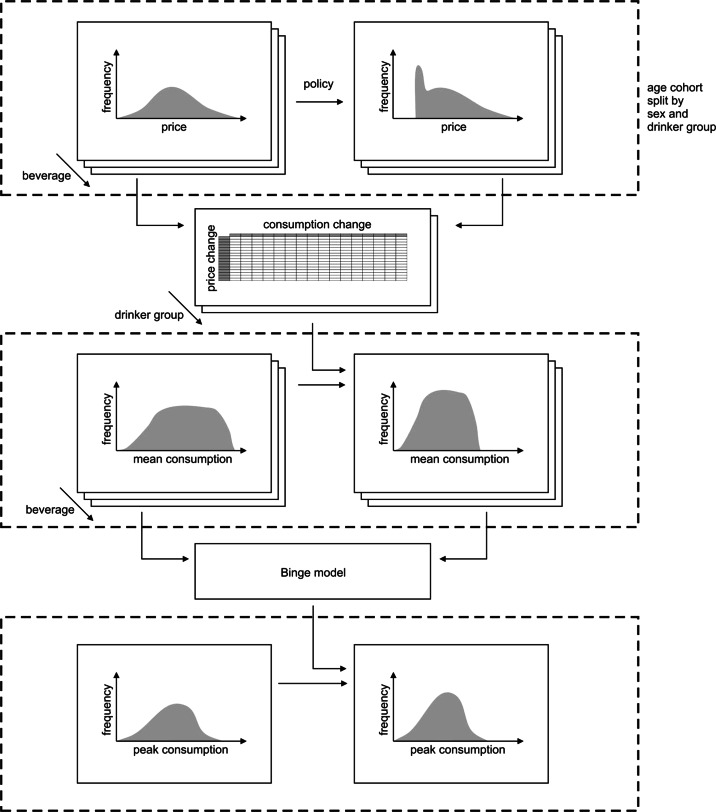
Policy-to-consumption model schematic

**Fig. 3:**
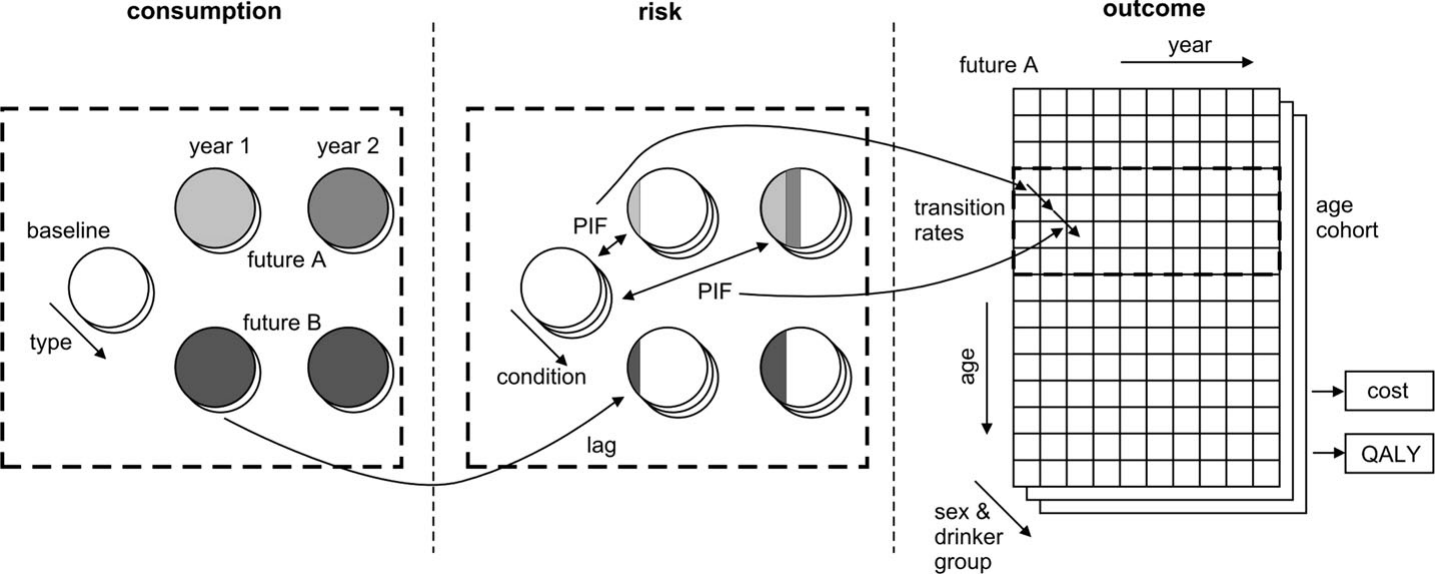
Consumption-to-harm modelling schematic

Figures 2 and 3 illustrate the sequence of modelling processes for policy to consumption and then consumption to harm. As an example, consider the effect of a 40 p per unit minimum price on the price and consumption distributions for the sub-group of 18-to-24-year-old male hazardous drinkers. Figure 2a illustrates that all transactions in the price data that are lower than 40 p per unit will be uplifted to the new proposed minimum 40 p per unit. The new average price across all (weighted) transactions will then be calculated for each beverage category and compared to the old average price to give the mean price increase. The vector of 16 mean percentage price increases for the sub-group will then be used in the econometric sub-model (the 16×16 matrix of own-price and cross-price elasticities in Fig. 2b) to produce a vector of 16 mean percentage consumption changes. These aggregate level consumption changes are then used to adjust the individual consumption records and create a revised distribution for the subgroup (Fig. 2c). For each individual used our linear binge models relating peak daily to mean consumption for each age/sex/drinker type group (Fig. 2d) to derive a revised peak daily consumption distribution for the subgroup (Fig. 2e).

Figure 3 illustrates how these revised mean and peak consumption distributions for the subgroup are then used to calculate harm changes via the risk functions and PIF approach. Figure 3a illustrates that the modelling of harm is formulated as a comparison of two possible futures in terms of consumptions, Future A versus Future B. In most applications of the model Future B consumption has been left at current baseline levels. The modelling begins with the baseline consumption distributions and looks forward 10 years in terms of consumption distribution trends for age age/sex subgroup. By far the most difficult aspect of model structuring concerned how to deal with various aspects of time passing in a coherent way, including the cohort ageing each year, the fact that there can be lags in harm effects, and that differential proportions of each subgroup die each year when comparing Future A and Future B.

Figure 3b illustrates that the revised absolute annual risk of mortality for each of the 47 health conditions is calculated each year for each age/sex cohort. Because there is a lag structure for the chronic conditions, the consumption distribution in year 1 affects not only year 1 risks but also those in year 2, year 3 and up to year 10. Thus in year 1 in Future A, the change in risk is due to 1/10th of full effect of the consumption distribution change between baseline and year 1. In year 2 in Future A, the change in risk is due to 2/10ths of the full effect of consumption distribution change between baseline and year 1, and 1/10th of the full effect of the consumption distribution change between baseline and year 2. Thus the changes in consumption over the years accumulate over time in terms of computing revised absolute risks of mortality. Not shown but also calculated is the revised absolute risk of disease prevalence for each condition for each age/sex subgroup, as measured by the proxy indicator of person specific hospitalisations for the disease.

Figure 3c illustrates how these revised annual risks are then applied to each population age/sex group cohort over time to calculate the survival, QALY and cost effects of consumption changes over time. The age-cohort approach models people in a particular age band (e.g. males aged 45–54), ignores the issue that this age band will be made up of different constituent members at different time points and assumes that the population distribution of drinking is stable over time within the age band. As model time progresses one year at a time, a calculation is done to quantify the estimated proportion of people in the subgroup who die within the model year from each condition. The difference between the numbers dying in Future A and Future B in the model year is calculated and the difference in QALYs lived is estimated by a life-table for the normal population adjusted for age/sex estimates of utilities for the average population. A second calculation is done to compute the prevalence of each of the conditions in the model yea, the QALY decrement due to being alive with the condition in the year and the associated in year treatment cost.

It is important to emphasise that the model is not a micro-simulation, operating on individuals within a cohort. The individual level data are merely used to describe non-parametric distributions for a sub-group. Effect sizes in most applications of the model to date are sub-group averages, assuming the entire sub-group has been impacted by the same percentage change in consumption. This assumption can be relaxed within the model (e.g. when modelling screening and brief interventions, only the proportion of the subgroup receiving an intervention had its the consumption distribution adjusted, Purshouse et al. 2009).

## Results

### Results framework

Model results can be presented in substantial detail for a single policy. Table 1 illustrates results for a 50 p minimum price per unit policy for subgroups of hazardous drinkers aged 18–24, moderate, hazardous, and harmful drinkers of all ages, and the whole population. Results for consumption levels indicate baseline consumption, mean reduction in consumption, and a split by beverage type, so that policy-makers can see which subgroups and types of alcohol are most affected in both relative and absolute terms. Results for purchasing indicate the mean change in spending per year for different subgroups, both on-trade and off-trade, together with the effects on changes in government (VAT and duty) and retailer revenues. For health, crime and workplace harms the model results can show details on the volume of annual incidents e.g. reduction in violent crimes or reduction in deaths due to road accidents annually, and these can be summarised at broader levels such as acute/chronic disease mortality and hospitalisation rates. Monetary costs to the health service and criminal justice system are shown. QALYs gained by the policy are shown, both health and crime related. Workplace harms and their monetary valuations are also shown, again allowing policy-makers to examine how these estimated effects compare between say moderate, hazardous and harmful drinkers. In moving towards a partial cost-benefit analysis approach, the monetary valuation of the harm reduction across health, crime and workplace harm is combined, allowing policy makers an indication of the relative effects across these three sectors and a summary total.

**Table 1:** Model results for a 50 p minimum price per unit policy scenario

			Hazardous 18–24	Moderate all ages	Hazerdous all ages	Harmful all ages	England total
*Consumption*							
Mean consumption per person per week			−0.70	−0.18	−1.49	−7.05	−0.84
Mean consumption per drinker per week			−0.70	−0.22	−1.49	−7.05	−1.05
% Change in mean consumption			−2.6 %	−3.8 %	−5.4 %	−10.1 %	−6.7 %
Baseline mean weekly consumption (units per person)			27.10	4.67	27.35	69.70	12.63
Change in volume of consumption (units per drinker per year)	On-trade	Beer	−43.80	−4.34	−54.49	−278.47	−37.80
		Wine	−16.37	−7.20	−27.18	−82.98	−17.60
		Spirit	−30.73	−4.78	−35.12	−114.11	−20.33
		RTD	0.09	0.00	0.00	−0.40	−0.03
	Off-trade	Beer	49.21	4.77	37.81	106.05	20.28
		Wine	0.46	0.00	0.56	0.56	0.17
		Spirit	3.15	0.22	0.60	1.06	0.37
		RTD	1.32	0.07	0.21	0.73	0.15
*Purchasing*							
Baseline value of purchasing (£per drinker per year)			£1,538.59	£275.66	£1,070.38	£2,447.79	£633.77
Change in value of purchasing		Off-trade	£13.21	£6.60	£35.62	£67.37	£17.87
(£per drinker per year)		On-trade	£69.88	£6.46	£47.21	£127.92	£25.30
		Total	£83.09	£13.06	£82.82	£195.29	£43.17
Total change in retailer revenue		Off-trade	£17.92	£157.84	£319.58	£305.15	£784.33
(£m after duty & VAT)		On-trade	£37.01	£93.30	£232.59	£226.39	£553.13
		Total	£54.93	£251.14	£552.18	£531.54	£1,337.47
Total change in VAT & duty received (£m)			£3.97	£1.15	−£2.97	−£65.17	−£66.93
*Health Conditions and Health Services*							
Deaths due to alcohol related diseases (per annum)		Chronic	−1	−27	−967	−1,759	−2,754
		Acute	−1	−89	−140	−76	−306
Alcohol related hospital admissions (per annum)		Chronic	−98	−9,202	−21,795	−52,017	−83,025
		Acute	−157	−4,562	−6,419	−3,611	−14,633
Monetary costs (£m)			−1	−57	−100	−144	−302
QALYs gained by policy			82	4,615	7,664	10,563	22,859
*Alcohol Related Crime*							
Cange in the number Offences (per annum)			−4,068	−3,694	−17,381	−17,799	−42,523
Costs (£m)			−6	−6	−20	−21	49
QALYs gained by policy			79	87	308	350	774
*Unemployment and Absences*							
Unemployment (per annum)		Volume	0	0	0	−25,905	−25,905
		Cost (£m)	0	0	0	−631	−631
Work absence (per annum)		Volume	−26,622	−89,813	−171,150	−179,457	−442,273
		Cost (£m)	−2	−9	−16	−18	−44
*Total Costs*							
Total change in health care costs + value of QALYs (£m)			1	36	53	67	156
Total change in crime costs + value of QALY’s (£m)			−7	−8	−26	−28	−64
Total value of changes to employment (£m)			−2	−9	−16	−649	−674
Total (£m)			−8	19	11	−611	−583

Model results can also be summarised to compare the effectiveness of different policies. Table 2 compares summary results for 18 different policies including general price rises, prices rises targeted only at low-priced products, minimum prices, discounting restrictions or bans, and what-if effects of advertising, outlet density or licensing hours restrictions. This allows policy-makers to compare policies in terms of effects on consumption, purchasing, revenues and harm reductions including proportionality of effects across subgroups. Policy-makers can then begin to see for example that a low threshold minimum price of 15 p or 25 p per unit has small effects, that the effects of a minimum price threshold accelerate as it is increased because a greater proportion of the market is affected, that banning discounts over 50 % (buy one get one free offers) has minimal effects, and that all of these policies affect harmful drinkers differentially from moderate drinkers. Detailed discussion of different aspects of these results and their implications for policy can be found elsewhere (Meier et al. 2005; Purshouse et al. 2009, 2010b).

**Table 2:** A comparison of modelled outcomes for a range of policies

Policy scenario	Change consumption (%)			Change in purchasing (%)			Change in purchasing (£m)		Change in harm (per year/after 10 years)			Change in harm spending (£m)		
	England total	Moderate drinkers	Harmful drinkers	England total	Moderate drinkers	Harmful drinkers	Retailer revenue	Duty + VAT revenue	Deaths	Health QALYs	Crime QALYs	Health	Crime	Employment
General price +10 %	−4.2 %	−3.5 %	−4.5 %	5.7 %	6.7 %	5.2 %	£1,051.70	£4.54	−1,517.77	−12,366.65	−1,621.66	−£161.65	−£98.15	−£337.73
General price +25 %	−10.9 %	−8.8 %	−11.7 %	12.2 %	15.4 %	10.8 %	£2,337.36	−£54.58	−3,785.61	−30,732.92	−4,143.21	−£401.87	−£250.43	−£853.33
Low priced off trade products +25 %	−0.9 %	−0.5 %	−1.4 %	1.6 %	1.0 %	2.0 %	£284.91	£8.40	−524.80	−3,812.94	5.79	−£49.72	−£0.77	−£77.97
Low priced on trade products +25 %	−0.6 %	−0.6 %	−0.4 %	1.4 %	1.6 %	1.5 %	£249.35	£17.30	−133.71	−1,370.61	−718.04	−£19.72	−£48.75	−£13.87
All low priced products + 10 %	−0.6 %	−0.5 %	−0.7 %	1.4 %	1.1 %	1.6 %	£239.14	£14.57	−265.30	−2,092.39	−288.34	−£27.94	−£20.03	−£37.33
Minimum price 15 p (off and on trade)	0.0 %	0.0 %	0.0 %	0.1 %	0.1 %	0.2 %	£21.47	£3.54	−0.88	−5.18	21.09	−£0.30	£1.11	−£1.53
Minimum price 25 p (off and on trade)	−0.1 %	0.0 %	−0.5 %	0.8 %	0.4 %	1.2 %	£137.12	£19.79	−47.36	−330.03	64.40	−£5.36	£3.28	−£36.74
Minimum price 50 p (off and on trade)	−6.7 %	−3.8 %	−10.1 %	6.8 %	4.7 %	8.0 %	£1,337.47	−£66.93	−3,060.23	−2,2353.74	−773.53	−£301.50	−£49.01	−£674.35
Minimum price 70 p (off and on trade)	−17.5 %	−11.5 %	−22.7 %	10.2 %	8.7 %	10.6 %	£2,295.23	−£387.66	−7,262.95	−55,083.73	−2,333.82	−£734.57	−£144.62	−£1,338.66
Minimum price 40 p off and 100 p on trade	−3.4 %	−2.0 %	−5.5 %	6.4 %	4.8 %	7.6 %	£1,142.37	£54.97	−1,568.06	−12,081.34	−898.44	−£162.34	−£56.11	−£380.73
30 p minimum price beers only	−0.2 %	0.1 %	−0.9 %	1.0 %	0.5 %	1.3 %	£168.53	£25.98	−47.27	−349.84	10.34	−£6.11	£0.77	−£83.64
Ban off trade discounting if >50 %	0.0 %	0.0 %	0.0 %	0.0 %	0.0 %	0.0 %	£0.98	−£0.02	−2.34	−17.55	−0.02	−£0.23	−£0.01	−£0.29
Ban off trade discounting if >20 %	−0.8 %	−0.5 %	−0.8 %	0.6 %	0.5 %	0.6 %	£116.37	−£11.10	−334.69	−2,563.34	−95.93	−£32.73	−£5.91	−£56.75
Total ban off trade discounting	−2.7 %	−1.9 %	−3.0 %	2.0 %	1.6 %	2.2 %	£411.86	−£43.76	−1,163.53	−8,932.99	−401.54	−£115.34	−£24.40	−£210.58
Ban off trade discount if reg price <30 p	0.0 %	0.0 %	−0.1 %	0.1 %	0.1 %	0.2 %	£18.91	£2.38	−10.75	−80.44	−0.83	−£1.15	−£0.06	−£7.51
Total advertising ban	−26.9 %	−26.9 %	−26.9 %	−26.9 %	−26.9 %	−26.9 %	−£3,316.38	−£1,708.74	−8,233.53	−69,551.49	−10,345.33	−£926.52	−£620.79	−£1,828.96
10 % reduction in outlet density	13.2 %	11.5 %	14.1 %	9.8 %	6.7 %	12.4 %	£1,114.85	£715.02	5,219.57	42,560.96	4,454.37	£559.03	£256.34	£1,217.50
10 % reduction in licensing hours	−1.2 %	−1.2 %	−1.2 %	−1.2 %	−1.2 %	−1.2 %	−£146.49	−£75.48	−417.42	−3,463.39	−466.20	−£45.32	−£27.96	−£94.07

One way and multi-way sensitivity analyses have been undertaken (Purshouse et al. 2009). Most were straight forward parameter estimate changes e.g. changing the time lag assumption for chronic health harms from a basecase 10 years to either 5 or 15 years to see the effects on results. The most complex of the sensitivity analyses have related to the structural form of the price elasticity estimates and the data used to derive them. Five examples are discussed here. First, basecase elasticity matrices were estimated for two groups, moderate drinkers, and hazardous and harmful drinkers combined, but other published evidence suggests there may significant differences between age/sex subgroups too. We undertook a sensitivity analysis attempting to account for this by weighting the cross-price elasticity estimates based on the difference between the preference vector of the particular subgroup and the preference vector for the aggregated groups. Second, for some age/sex subgroups the EFS purchasing data does not provide a good match when compared with the GHS consumption data, partly due to purchases by one person being consumed by another, so we reallocated purchases where this seemed plausible and obtained an improved match between the EFS and GHS consumption data. Third, the elasticity estimates derived using the econometrics model showed heavy drinkers as slightly more responsive to price change than moderate drinkers, and we carried out a sensitivity analysis to explore the changes to the model results if heavy drinkers are assumed to be one third less responsive than moderate drinkers (Chisholm et al. 2004). Fourth, we undertook a set of analysis using completely different, previously published long run UK elasticity estimates from Huang (2003) based on high-level time series data, in which both own-price and cross-price elasticity estimates are greater than those derived from EFS. Finally, probabilistic sensitivity analysis was used to explore the impact of uncertainty in the coefficients of basecase elasticity estimates from the regression using Cholesky decomposition, with the results suggesting that the parameter uncertainty around these coefficients is much less significant that the structural assumptions described above.

In terms of effects on results, we found fairly tight confidence intervals for changes in alcohol consumption given the uncertainty in cross-price and own-price elasticities. For example, for a 40 p minimum price policy the confidence interval for change in alcohol consumption was −2.4 % +/−0.2 % (Purshouse et al. 2009). A re-estimation of the elasticity matrices having made some adjustments to reflect concern that some alcohol purchased by females in the EFS was actually consumed by males in the household did produce a slightly larger effect (−2.7 %). Using alternative published evidence on elasticities also had slightly stronger effects e.g. long run estimates from HMRC (−2.2 %), or a modelling assumption which reduces the elasticity estimates for hazardous and harmful drinkers by one third (−2.0 %). Other sensitivity analyses showing relatively small effect included: different slopes for the expected scale of binge given mean consumption function, the exclusion of any protective effects of alcohol, alternative time to full effect for chronic harms ranging from 5 to 15 years, use of alternative evidence on the multiplier for the extent of reporting of “less serious wounding” crimes and on the fraction of crimes attributable to alcohol, use of UK-based work absence data, use of a lower value for salary to compute unemployment effects, and the value for the relative risk of not working for harmful drinkers Each had some small or modest effect (+/−25 % of the basecase for 10-year cumulative value of harm) except for the relative risk of not working for harmful drinkers (+68 %). All of these sensitivity analyses were on overall model parameters which do not specifically affect any one policy over another and therefore do not substantially affect the relative rankings of policies. More detail is given in the report to NICE (Purshouse et al. 2009).

Three main approaches to validity testing for the modelling were undertaken. The first was functional testing of the Microsoft Excel VBA implementation of the model including double checking spreadsheet structures, VBa code, counts and sum totals, and also testing that changes in parameters have the expected scale and direction of effect in subcomponents of the model. Secondly, we cross-checked the modelling outputs for a counterfactual scenario of zero alcohol consumption in the population of England against the published estimates of the burden of attributable harm, including those for mortalities, NHS costs, overall crime, and the total financial valuation. These analyses showed the model produced results with the same order of magnitude for alcohol attributable harm measures in each case. Thirdly, we crosschecked some of the emergent model parameters (e.g. overall price elasticity) against published literature estimates. For example, the own price elasticity estimates were compared against those in the recent systematic reviews and meta-analyses by Gallet (2007) and Wagenaar et al. (2009) which found, respectively, a median elasticity for alcohol of −0.535 and a mean elasticity for alcohol of −0.51, as compared with an overall effect within our model for a 1 % general price rise of around −0.42.

## Discussion

### Relationship between the modelling and policy debates in the UK

In the UK, this work has been critical to a series of policy debates since the initial report for the Department of Health was written. In England, a weak form of minimum pricing has now been introduced, stipulating that no product can be sold below the cost of alcohol duty plus VAT. There has also been a ban on some ‘irresponsible’ discounting practices in the on-trade sector. A key discourse, driven by industry stakeholders and parts of the media, has been around whether price policies penalise low income drinkers (“punishment of the poor”). Other research groups have sought to provide counter-evidence (Record and Day 2009; Ludbrook 2010). Version 3 of the model will also include explicit modelling of the effects on different income groups. In Scotland, an initial attempt to introduce a 45 p minimum unit price was narrowly defeated in parliament, when introduced by a minority government. Policies restricting price discounts were however approved. In the most recent election in Scotland, the governing party now has an overall majority and a minimum price is back on the political agenda. The key challenge for such a policy remains the demonstration of ‘proportionality’ of the pricing policies as defined under EU law, i.e. that the degree of interference in the common market is necessary to avoid significant harm. Detailed health economic models such as ours are ideally placed to provide relevant evidence on which such decisions can be based.

The role of the analyst in such policy debates can be complex. How should the analyst engage in dissemination of the work? Should the analyst take into account or engage in activities related to the obstacles to policy implementation? The traditional OR analysts’ position is one which (most of) the team would generally hold i.e. that the analyst analyses to support the decision makers in their difficult decisions. In the case of a minimum unit price for alcohol, the decision makers in the end will be members of parliament, and hence different perspectives and political philosophies are also at play. Throughout the evolving twists and turns in the debate, we have been keen to provide analysis and information that informs the argument. We thus worked hard to enable the model to examine moderate drinkers, which, it became apparent, would be part of the decision makers’ considerations. More recently, the shift to quantifying separate effects for low income drinkers have been requested and are being worked through. In a sense, this is at most a tweak to the traditional analyst position because it is merely being extra responsive to decision maker needs. Slightly more complicated is the fact that we have found ourselves ‘in the debate’ as part of our process of research dissemination. This has involved media interviews, radio and television appearances, all wanting short but clear descriptions and understanding of the work’s findings, especially on the potential scale of impact of different policies. To an extent, we have found that journalists have automatically positioned us as ‘pro’ the policy. Some supporters and opponents of the policy have also positioned us as pro the policy and some opponents have attacked our work. We have been careful to remind journalists, and supporters and opponents of the policy, that our role has been to analyse data and evidence to estimate as accurately and fairly as possible the potential scale of effect of different policies. In responding to attacks on our work, we have sought to take the same approach that we do when peer reviewers provide comment and critique—a careful and considered response, directing people to the relevant evidence, its limitations and the conclusions we can have drawn.

### Scope and monetary valuation issues

There are extended challenges in applying economic modelling to macro-level interventions, beyond those commonly encountered say in NICE health technology assessments. In particular, the range of costs and benefits to be included can be difficult to determine, especially when decision-maker and stakeholder concerns may not be limited to the immediate and direct effects of an intervention. Direct Policy implementation costs, which we define as the direct financial costs to government of legislative processes, staff time for implementation and enforcement through existing mechanisms, for regulation of alcohol prices, advertising, outlet density or licensing hours are likely to be minimal and as such we have to date excluded these from the detailed analysis. Valuation of health and crime harm reductions have been estimated using a quality adjusted life years gained framework (to patients and victims respectively), with a financial value for a health-related QALY and a crime-related QALY applied. Valuation of workplace harm reductions, i.e. sickness absence and unemployment, were quantified financially based on average salaries.

Some might argue for a purely public sector stance to be taken by decision-makers, whereby for example, larger price increases produce greater estimated harm reductions with relatively small public sector implementation costs i.e. ever larger price increases would be considered more ‘cost-effective’. From a public sector perspective some might argue that costs of the lost productivity from public sector employees should be included and also possibly any government costs relating to sickness and unemployment benefit payments for the whole population. There is some debate about the latter costs, since it could be argued that these should be treated as transfer payments (a redistribution of income in the market system which does not directly absorb resources or create output) and therefore be excluded. At present we have examined workplace costs for the whole population, not separating public sector from non-public sector.

Costs to individuals (either drinkers themselves or to their family, friends and colleagues) were outside the scope of the NICE economic assessment, although they may be considered in terms of equity implications. In the original analyses for DH, we analysed increased expenditure by consumers. Such direct effects were included at the request of policy makers. For retailers, the model produces estimates of changes in volumes of alcohol expected to be sold as a consequence of each policy, which are then combined with price information to derive, for the country as a whole, the retail sales value (£) of different types of alcohol in both the off-trade and on-trade. These estimates are not broken down by type of retailer or particular named retailers. Nor do they make any estimates of profit or otherwise from alcohol for retailers since analysis of retailers’ cost-base are not included in the modelling. Similarly, there is no quantified assessment here (beyond the retail sales overall) of the potential impact on different producers of alcohol, since direct information on their costs, the wholesale market, and the profit made by producers in selling on to retailers are not covered by the modelling.

Some other transitional costs are not examined here, including effects on the advertising or media industry. It is important not to misinterpret the increased costs to consumers and increased sales values to retailers: the changes in consumer expenditure under the different scenarios are not ‘net effects’ and cannot be interpreted as ‘costs of the intervention’ against which the ‘savings of the intervention’ (e.g. in terms of public sector health and crime or wider workforce savings) should be balanced. This is because the increased expenditure by consumers has to be considered in conjunction with the increased revenue to the alcohol industry (producers, wholesalers and retailers) and possibly reduced revenue to other sectors of the economy. The increased revenue to the alcohol industry will return to the wider economy in a variety of ways; for example, wages and salaries to industry employees, profits to individual and institutional shareholders, including pension funds, and potential price reductions on other goods where retailers have been using alcohol as a loss leader. The analysis presented here does not include this dynamic analysis of the full effects of redistribution through the economic system.

Expected changes in tax revenue income to government were also modelled. Again these are not ‘net effects’ and were included for information, rather than for direct trade-off calculations in relation to public sector benefits. If increased revenue were to accrue to the Treasury, then this also can be conceived of as returning to the wider economy in the form of increases in government services or reductions in other taxes. The public sector focus of NICE economic evaluations also excluded consideration of welfare losses (typically defined by consumer surplus as an economic measure of consumer satisfaction based on the difference between the price of a product and the price a consumer is willing to pay) arising from reduced consumption of alcohol. Consumer welfare analysis has not been undertaken as part of this study. Such an analysis would need to account for potential increases in consumer surplus from any price reductions elsewhere in the economy and the problems of estimating a ‘pure’ demand curve for alcoholic beverages.

### Limitations due to data and evidence gaps

Ideally, price analyses would be based on longitudinal data recording both alcohol consumption and purchase. Such data is not available in the UK and we chose to use the annual cross-sectional EFS purchasing data which provides information on alcohol volumes and prices paid at an individual level. However, it may not be appropriate to assume that the consumer is the purchaser (i.e. if alcohol purchased by one household member is then consumed by a different household member). The current econometric model, although it does allow some of the complexity of the problem to be modelled, remains a relatively simple approach, especially with zero purchase observations (people who did not buy alcohol during the 14 day diary period) not modelled separately from non-zero observations. A new longitudinal survey obtaining both price and consumption data would be very valuable in such a context.

Peak consumption was estimated from the GHS, in which respondents were asked how much they consumed on the heaviest drinking day in the past 7 days before the interview. We then used this as a proxy for binge drinking. Binge drinking would be better represented by a combination of both frequency of heavy drinking occasions and average consumption level on such occasions, if such data were available.

General population surveys, including EFS and GHS, are known to underestimate population level alcohol expenditure and consumption levels by around 40 % because of underreporting, and miss or under-represent population groups at risk of alcohol-related harm, such as the homeless (Stockwell et al. 2004). A recent study proposed a statistical method to shift survey consumption to population level sales data to account for this underestimation (Rehm et al. 2010). Further research is needed to address this important issue.

We assumed no delay between price-based policy implementation and price changes, effective enforcement/full compliance of supply-side with the policy and that only targeted products would be affected. For example, in our model a minimum price for spirits does not lead to price changes in products other than spirits. This assumption ignores market response to price changes. Research analysing the supply-side response to pricing policies is desirable, because it is plausible that policies that have a large effect on beverage prices might lead to market restructuring, and supply-side responses are unlikely to be straightforward (Kenkel 2005; Young and Bielinska-Kwapisz 2002).

In terms of harms included, one can conceive of the hospital admissions, and associated costs, as a proxy for alcohol associated morbidity. Over the longer-term, we implicitly assume that thresholds for hospital admission do not change over time, but of course there may be changes in treatment options, available hospital capacity, clinician preferences etc. which could affect hospital admission rates for different disease whilst being less related to an individual’s harm. To a degree this may also be true for police related activity and crime.

We assumed a linear time lag between a change in consumption and a change in mortality and morbidity risk for all chronic illnesses, however, different illnesses may have different lag structures (e.g., liver cirrhosis vs breast cancer) and the change of risk may be the same each year. Further systematic review and analyses would help identify disease specific and more sophisticated time lag structure and improve the understanding of the relationship between changes in consumption and changes in risk, for different types of harms. We also assumed that a type of harm is either related to mean consumption or to peak consumption, however, some types of harm may be caused by a combination of mean and peak consumption (e.g., suicide, heart disease). There is also no consensus on the threshold above which risk begins to increase, especially for acute harms, the current model assumes 32 g/24 g per day for acute harms and 24 g/16 g per day for chronic harms, for male and females respectively. In general, risk modelling is better developed for health harms than for crime and workplace outcomes, especially unemployment. Further research is needed to examine what proportion of these outcomes is attributable to alcohol.

The current model is able to appraise a wide range of price-based policies including general price changes, minimum pricing and some policies affecting availability measures and advertising restrictions. However, the evidence on the effectiveness of price-based policies is more robust and abundant than for availability measures. Further research to examine the effectiveness of availability measures and potential policy mix (e.g., simultaneously introducing availability and price-based policies) would be useful.

We used 2006 data for alcohol consumption and purchasing as the baseline and did not consider underlying trends, thus implicitly assuming steady-state alcohol consumption in the “do-nothing scenario”. We believe that analysing the difference between strategies over time is reasonable using the steady state model in this way because, for the most part, trends in consumption would be occurring equally in both arms of the comparison. It is *more* challenging to validate the current model against historical data because of other factors affecting alcohol consumption, such as changes in disposable income and licensing hours, arise simultaneous to price changes. Further analyses to identify the underlying trends in alcohol consumption and further development of the model to establish a dynamic ‘do-nothing scenario’ will *further test* the credibility of the model and facilitate model validation.

In terms of recommendations to government or other agencies in the UK, or internationally, our single biggest priority would be to attempt longitudinal data collection on a population representative sample of people, collecting both data on their consumption patterns and their purchasing including types of drinks, place of purchase and prices paid.

### Reflections on our approach and planned further developments

Through several parts of the research process, the research team has reflected on our practice. The engagement with stakeholders/policy-makers/funders has been important in this, as has the formal peer-review of research reports and subsequent journal articles, and peer-review of proposed further research. In the main, the research team feels that the technical work achieved through the model development has been strong and fit for purpose. Where we believe more time for further sophistication in the data analysis/modelling, or further primary data collection/secondary review of evidence would benefit decision makers, we have said so in the limitations and proposed further work. The sections above on limitations reflect such technical areas. If asked “would the authors do anything differently if they could re-do the model afresh?”, then given the same circumstances and evidence, we feel the answer would be that we would make many of the same decisions in our modelling processes. Priority technical areas to further develop have been the econometrics for price elasticities, and more sophisticated analysis of datasets on patterns of risky single occasion drinking. From a software perspective, writing the original model in EXCEL/VBa has produced several problems when considering extension of the model to other research questions, and we are now moving to a more modular and traditional programming language approach using Visual Basic, which allow more flexible reporting of results, and including some software development processes including the concept of “Use cases” into the project planning. An option we have considered several times, and not yet taken forward, is to generate a simulated integrated dataset for the alcohol consumption and purchasing of every individual in England, calibrated to match with the GHS and EFS datasets for consumption and purchasing respectively.

We are now working on a 3-year project funded by the Medical Research Council in the UK to further develop the model and enhance the understanding of the effectiveness of alcohol public health policies. The key areas of development include (1) primary research into supply-side responses to tax and price changes in the UK; (2) systematic review of the relationship between heavy drinking occasions (binge drinking) and harms; (3) systematic review of time lags from policy to consumption and from consumption to harms; (4) the development of new econometric models to address the limitations of the current method; (5) systematic review of policy context and its impact on policy effectiveness; (6) enabling appraisal of availability policies and policy mix; (7) development of a new dynamic model, which not only incorporate the findings of the rest of the project work packages, but also accounts for trends in alcohol price, income, consumption and harm.

Beyond this work, we plan as part of our wider research programme to engage with decision makers at Local Authority level in England to develop and test a localised version of the model to support their policy analysis, to work through evidence sources on the harms done to others e.g. partners, families, in the workplace etc. through heavy drinking, and to begin to develop a joint alcohol and tobacco version of the model.

### Adaptability of the framework to other questions

Since the original framework was developed we have engaged in adaptations which have updated the analysis and results as new survey data has become available. Within England, the framework can be fairly easily applied to any intervention where there is an effect on alcohol consumption levels, for example, we have been able to adapt the model to produce estimates of the cost-effectiveness of screening and brief interventions for alcohol in a variety of different settings (Purshouse et al. 2009).

Country adaptations of the model have also been undertaken and further adaptations are ongoing. Adapting to Scotland (Petra Meier et al. 2009) required use of different but very similarly structured datasets on baseline consumption, mortality, hospitalisations and crime. We are in the process of adapting the minimum pricing modelling to two provinces in Canada, and the screening and brief intervention modelling to Netherlands, Italy and Poland, all of which seem at this stage to be feasible. The key issue in these the adaptations is data—on consumption, mortality, hospitalisations, crime and employment. In each case, a process of developing a detailed understanding of the datasets available in the country concerned has been essential, and this, when followed by some brainstorming with experts on the consumption, epidemiological or health economic data concerned, has resulted in minor restructurings to the model software (of the order of a few days) and use of agreed assumptions with our collaborating researchers to produce an adapted model. Having undertaken 5 such country adaptation exercises, we are beginning now to investigate a more streamlined adaptation approach using a meta-model. This plan would essentially run the England model at a reasonable number of design points for key variables and compute key summary outputs, thus enabling the key differences between England and the target country for adaptation to be modelled.

Adaptation of the generalised framework of the model (i.e. incorporating details of purchasing and behaviours from survey data sets with epidemiological models of risk) to model broader problems than just alcohol is also conceivable. The consumption and purchase of food in relation to policies and tax on foodstuffs and related risks of unhealthy diets, obesity etc. could plausibly be modelled, as could purchase of tobacco and the harms due to smoking, or potentially even the incentivising of physical activity. We believe that our model framework can provide a useful reference point for such single behaviour public health policy models. An important extension to the work would be modelling the interaction of multiple behaviours rather than a single one. For example, there are known correlations between drinking alcohol and smoking (Pierani and Tiezzi 2009), and the inter-relationship is complex when policy impacts are to be modelled because different policies might have different consequences e.g. increasing alcohol prices might cause people to substitute instead to more tobacco, the pub smoking ban could cause people to drink less because they visit the pub less, or more because they decide to smoke instead at home and drink more of the cheaper alcohol. An even wider multiple behaviour model could be conceived incorporating alcohol, smoking, diet and physical exercise, and though we are currently engaged in such a research project modelling the joint behavioural impact of health messages to students starting University, it is unclear at this stage whether such a detailed level of granularity of behaviours as undertaken in our Sheffield Alcohol Policy model, would be the most feasible or efficient approach.

## Conclusion

In conclusion, we have attempted to develop a general framework for alcohol policy modelling.

In terms of the wider policy analytics agenda, this work has been directed by requests from government departments (DH and Home Office) and agencies (NICE) and has managed to incorporate systematic use of data, evidence, OR methods and leading-edge policy modelling. The data used is principally from large-scale surveys, which have be used to quantify baseline alcohol consumption, price elasticities for alcohol via econometric modelling, and quantified relationships between levels of (mean and peak) alcohol consumption and attributable harms in three domains: health, crime and workplace. A broad valuation of harms analysis, applying financial costs to each type of harm, enables some estimate of total financial value of the harms avoided by different policies. It has principally used operational research methods of problem structuring, modelling and evaluation.

The work has been used interactively with government and its agencies at different phases of the policy cycle including issue identification, policy testing and refinement, and pre-implementation policy analysis/evaluation. It has also, through the process of detailed publicly available reports, and formal government consultation exercises, been a key part of the public debate, providing a level of transparency for the public and stakeholders in industry, health, crime and other sectors to comment upon.

We hope the framework will develop further as research progresses and that it might be generically useful to the evaluation of public health strategies in areas other than alcohol.

## Footnotes

1 NICE also examined alcohol screening and brief intervention strategies (see Purshouse et al. 2009).
